# Successful removal of a migrated duodenal-jejunal bypass liner from deep jejunum via single-balloon enteroscopy

**DOI:** 10.1055/a-2845-1803

**Published:** 2026-04-20

**Authors:** Chao Huang, Jun Li, Feng Liu

**Affiliations:** 1 Department of Gastroenterology, Shanghai Tenth People’s Hospital, Tongji University School of Medicine, Shanghai, China

**Fig. 1:**
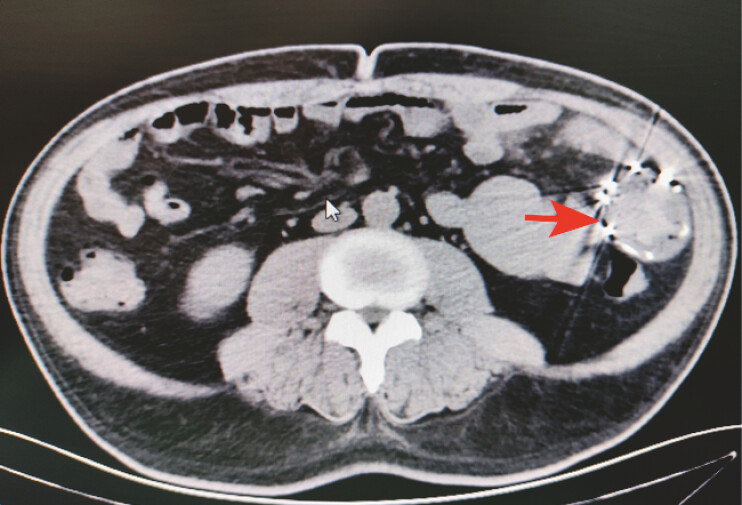
A computed tomographic image showing an metallic stent-like high-density shadow in the left mid-abdomen (red arrow).

A 46-year-old man presented with a 5-day history of melena with a dropped hemoglobin of 71 g/L. Computed tomography revealed a high-density shadow in the left mid-abdomen ( Fig. 1 ). He gave a history of duodenal–jejunal bypass liner (DJBL) implantation for weight loss 4 months ago. Emergency gastroscopy and colonoscopy were performed and bleeding in the stomach, duodenum, or colorectum was ruled out, leading to a clinical judgment of DJBL migration to the deep small intestine. After a multidisciplinary evaluation, the endoscopic retrieval of the DJBL by single-balloon enteroscopy was attempted.

**Fig. 2:**
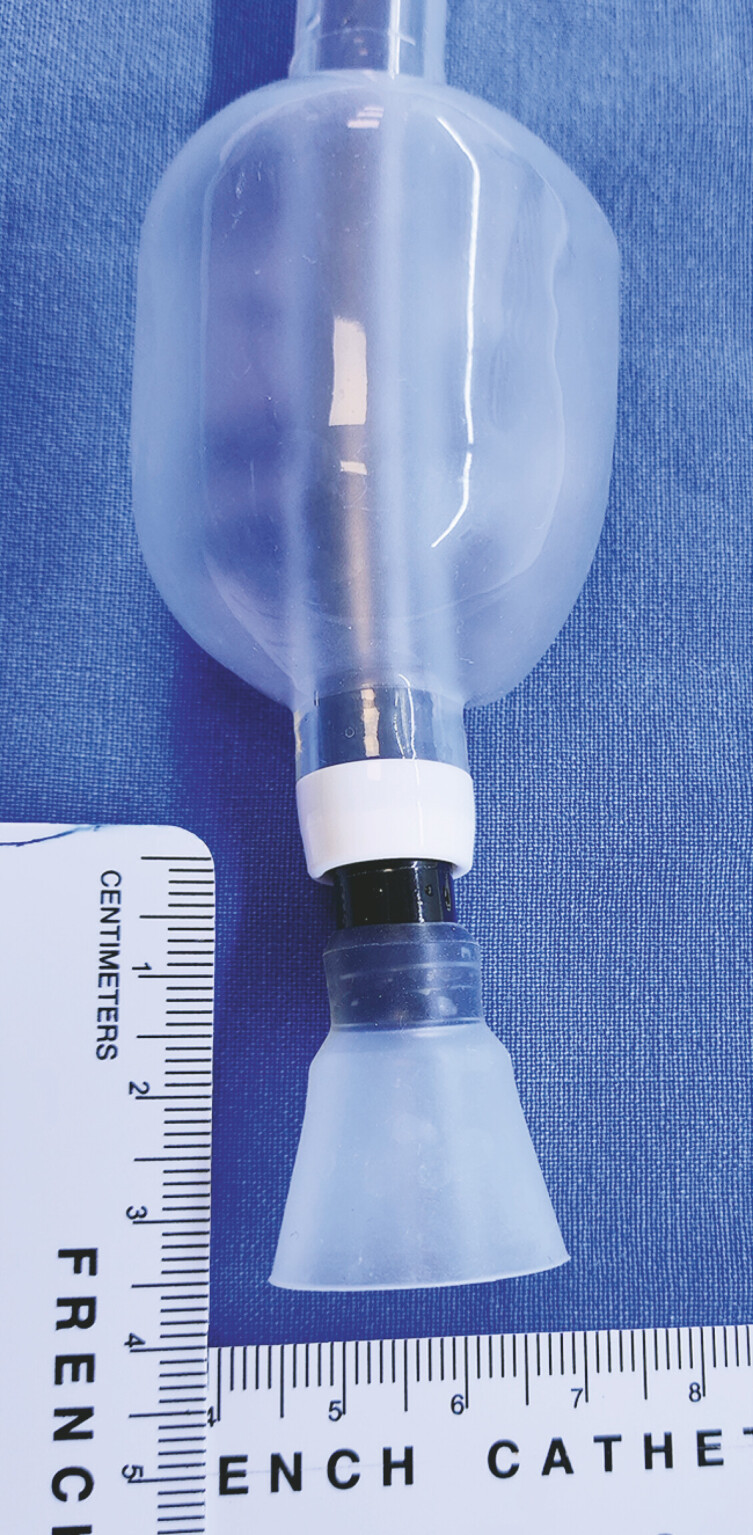
A horn-shaped silicone cap was equipped at the front end of the enteroscope before the procedure which is necessary to avoid further mucosal injury from the DJBL barbs. DJBL, duodenal–jejunal bypass liner.

**Fig. 3:**
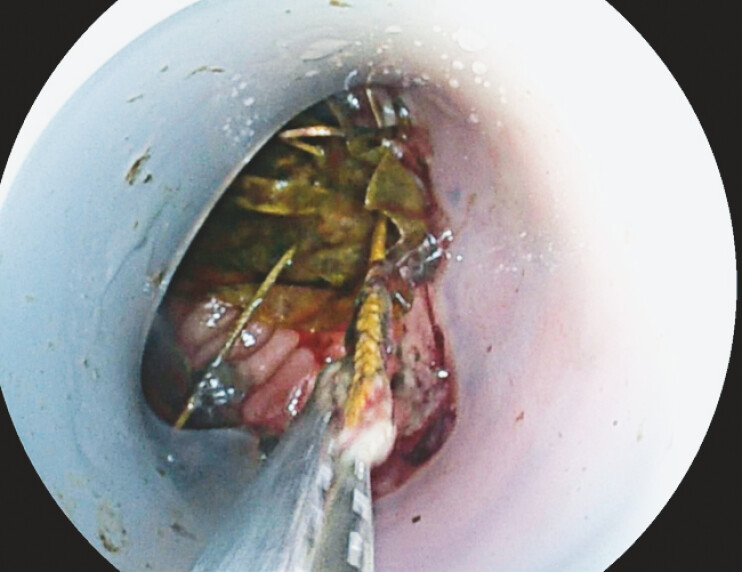
An elongated foreign body forceps was used to grasp the retrieval string. Make sure that all the barbs were encased by the silicone cap to prevent laceration and perforation during retrieval.

**Fig. 4:**
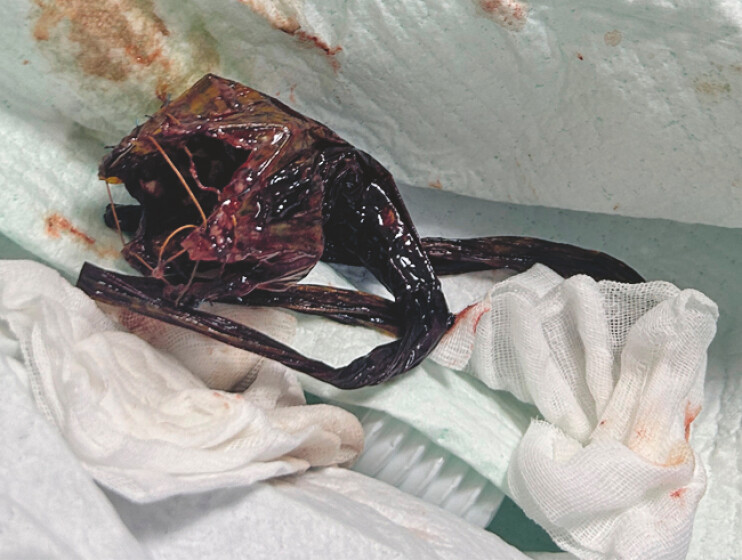
The intact DJBL was removed out of patient’s body. DJBL, duodenal–jejunal bypass liner.

A horn-shaped silicone cap was equipped at the front end of the enteroscope ( Fig. 2 ). Upon advancing the enteroscope to deep jejunum, the self-expanding anchor of DJBL was observed stucked in the lumen. The sharp barbs of the DJBL, which were designed to keep the DJBL in place, had embedded in the jejunal mucosa, causing multiple ulcers and bleeding. After thorough irrigation and suction, an elongated foreign object forceps was used to hook the recycling string ( Fig. 3 ). After all the barbs were retrieved into the silicone cap, the DJBL was removed successfully ( Fig. 4 and Video 1 ). A second enteroscopy ensured no active bleeding or perforation wherever the DJBL reached.

A migrated duodenal–jejunal bypass liner was successfully removed from the deep jejunum via single-balloon enteroscopy with the help of a horn-shaped silicone cap.Video 1

DJBL implantation is a novel, less-invasive procedure for the treatment of morbid obesity 1 . Device migration and gastrointestinal hemorrhage are two of the severe adverse events post-DJBL implantation and the distance of migration in most cases has not been clearly expounded 2 . In this case, the DJBL was located 2.5 meters posterior to the duodenal bulb, presenting the greatest migration distance that has ever been reported. To our best knowledge, this is the first reported case of the successful retrieval of migrated DJBL by single-balloon enteroscopy, which provides valuable experience for the minimally invasive treatment of complications related to DJBL migration.

Endoscopy_UCTN_Code_CPL_1AH_2AJ
